# Rupture Prediction for Microscopic Oocyte Images of Piezo Intracytoplasmic Sperm Injection by Principal Component Analysis

**DOI:** 10.3390/jcm11216546

**Published:** 2022-11-04

**Authors:** Naomi Yagi, Hyodo Tsuji, Takashi Morimoto, Tomohiro Maekawa, Shimpei Mizuta, Tomomoto Ishikawa, Yutaka Hata

**Affiliations:** 1 Advanced Medical Engineering Research Institute, University of Hyogo, Himeji 670-0836, Japan; 2 School of Engineering, University of Hyogo, Himeji 671-2280, Japan; 3 Reproduction Clinic Tokyo, Tokyo 105-7103, Japan; 4 Graduate School of Information Science, University of Hyogo, Kobe 650-0047, Japan

**Keywords:** oocyte, rupture, Piezo-ICSI, principal component analysis, support-vector machine

## Abstract

Assisted reproductive technology (ART) has progressed rapidly, resulting in a great improvement in the clinical pregnancy ratio. When applying the protocol of piezo intracytoplasmic sperm injection (Piezo-ICSI), it is very important to puncture the zona pellucida and the oocyte cytoplasmic membrane without rupturing the oocyte cytoplasmic membrane. Previous studies have shown that the poor extensibility of the oocyte cytoplasmic membrane might be closely related to rupture. However, no consensus has been reached regarding how the quality of the oocyte for extensible ability or rupture possibility affects the surfaces of the oocyte on the microscopic frames. We conducted this study to provide evidence that artificial intelligence (AI) techniques are superior for predicting the tendency of oocyte rupture before puncturing on Piezo-ICSI. To inspect it, we provided a retrospective trial of 38 rupture oocytes and 55 nonruptured oocytes. This study marked the highest accuracy of 91.4% for predicting oocytes rupture using the support-vector machine method of machine learning. We conclude that AI technologies might serve an important role and provide a significant benefit to ART.

## 1. Introduction

Recently, the Japan Society of Obstetrics and Gynecology, which investigates trends in assisted reproductive technology (ART), and the International Committee Monitoring Assisted Reproductive Technologies have reported that the number of individuals receiving infertility care in Japan has been increasing each year [1,2]. Intracytoplasmic sperm injection (ICSI) is an assisted reproduction technique [3] and consists of conventional ICSI and Piezo-ICSI, with the difference in how to puncture the membrane [4]. With conventional ICSI widely used, the tip of the injection pipette is sharp and pierces the zona pellucida of an oocyte and draws the oocyte cytoplasmic membrane. With Piezo-ICSI, the tip of the injection pipette is flat and punctures the zona pellucida and the oocyte cytoplasmic membrane without drawing the oocyte cytoplasmic membrane. Piezo-ICSI is the current established standard procedure and performed using small axial mechanical pulses with piezoelectric elements [5,6]. As compared with conventional ICSI, Piezo-ICSI has the representative disadvantage and includes: (1) difficulty to develop a system with consideration for the shape or the thickness of the injection pipette, (2) difficulty to control the stable piezo elements, (3) requires higher expenses. On the other hand, Piezo-ICSI has the advantage that the fine punctate vibration of a piezo-driven flat injection pipette allows for minimal distortion of the oocyte over conventional methods. This technology results in a high fertilization rate and low degeneration rate. However, during the puncturing process, the poor extensibility of the cytoplasmic membrane of the oocyte results in its rupture, causing degeneration [7,8]. In addition, there are many difficulties in predicting the possibility of rupture of the oocyte cytoplasmic membrane using only human visual estimation. Even well-trained embryologists recognize that it is difficult to judge membrane rupture for short-term events with the images before puncturing. The ICSI has a complicated procedure and it is reported that its standardization could contribute to a systematic review [9]. New techniques are needed to improve the success rate of ICSI more consistently by reducing human involvement. If the possibility of rupture can be suggested before puncturing, embryologists can carefully handle the puncture process. The integration between human technology and AI that exceeds human judgment will be useful in improving ICSI outcomes. For example, they face the problems that if the membrane is unintentionally ruptured, the oocytes are prone to degeneration. In addition, even if it does not denature after rupture of the membrane, the subsequent culture results will be poor. Therefore, we designed our study by applying image processing to establish evidence that the images before puncturing can predict the possibility of rupture of the oocyte cytoplasmic membrane.

Many human visual system models are being developed in the field of computer vision, which has pioneered artificial intelligence (AI) [10]. Several studies have previously addressed texture analysis based on feature distributions with local binary patterns (LBP) used for classification in computer vision [11,12,13]. A diagnostic-assisted tool was proposed in the field of ART, which is based on oocyte texture image processing [14]. Although currently AI applications in reproductive medicine [15] are being reviewed, the number of them is still rather small. We developed a support system to identify the optimal puncture position of the oocyte cytoplasmic membrane by applying LBP and investigated the mechanism of the endometrium in our previous works [16,17,18]. In addition, the computer intelligence technique was implemented for the first time and proved its efficacy in the field of reproductive medicine [19]. However, there is currently no evidence on the effects of the rupture of the oocyte cytoplasmic membrane on the image texture before puncturing. To our knowledge, this is the first trial investigating an association between the characteristics of a microscopic movie of oocytes before puncturing and the possibility of rupturing the oocyte cytoplasmic membrane. Our hypothesis was that the microscopic movie may include the potential to predict rupture. We conducted a retrospective clinical study to confirm this hypothesis.

## 2. Materials and Methods

### 2.1. Study Design and Oversight

The Institutional Review Board approved the protocol at the Reproduction Clinic Tokyo (1 September 2019; No. 2019-T-L-01) and the Graduate School of Information Science, University of Hyogo (28 April 2021; No. 202101). All subjects provided informed consent before enrollment. We conducted all study procedures in accordance with Good Clinical Practice guidelines and the Declaration of Helsinki. Subjects’ information was anonymized and deidentified before analysis.

**Figure 1:**
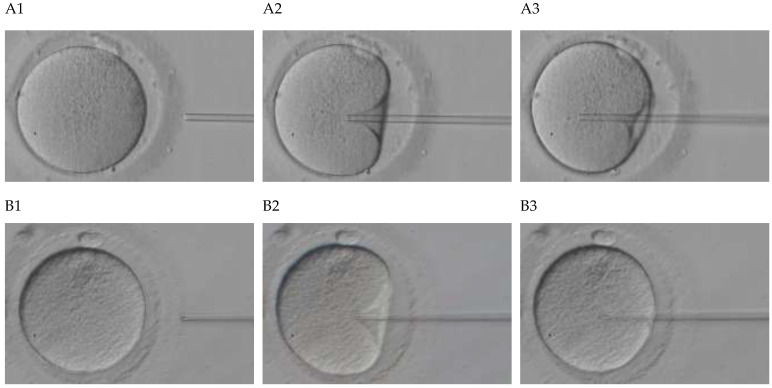
Oocyte under Piezo-ICSI. (**A**) Nonruptured oocyte. (**B**) Ruptured oocyte. **A1**, injection pipette directed toward the center of an oocyte. **A2**, injection pipette of **A1** was inserted more deeply. **A3**, the injection pipette of **A2** was inserted more deeply, and it smoothly penetrated the oocyte cytoplasmic membrane without rupture. **B1**, an injection pipette directed toward the center of an oocyte. **B2**, the injection pipette of **B1** was inserted more deeply. **B3**, the injection pipette of **B2** was inserted more deeply, and it did not smoothly penetrate the oocyte cytoplasmic membrane with rupture. All images for **A1**–**B3** are of 744 pixels width and 453 pixels height.

We retrospectively collected and enrolled 93 oocytes assigned to Piezo-ICSI at the Reproduction Clinic Tokyo in 2019. Based on the presence of membrane rupture during membrane extension, as shown in Figure 1, oocytes were classified into two groups: rupture (*n* = 38) and nonrupture (*n* = 55). Finally, we analyzed the microscopic movie using the LBP method and the principal component analysis (PCA) method and applied a support-vector machine (SVM) method.

### 2.2. Procedure Recording Protocol

**Figure 2:**
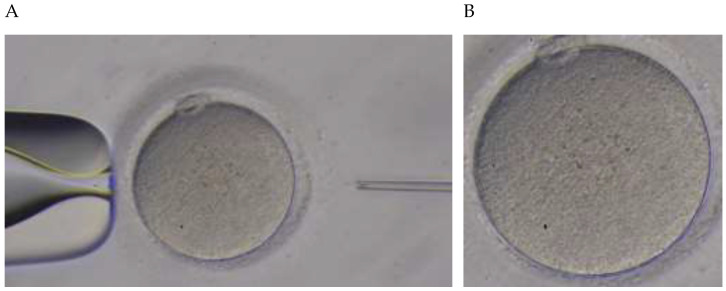
Image of oocyte. (**A**) Raw frame image of 960 pixels width and 540 pixels height, captured from microscopic movie. (**B**) Trimming image of 380 pixels width and 370 pixels height specified, based on the raw frame image A, is an analyzed target image.

During Piezo-ICSI, the puncturing process for the oocytes was individually recorded with a microscope as moving images. The details of the digital recording for the microscopic movie were indicated as codecs of Windows Media Video (compression coding format developed by Microsoft, Redmond, WA, USA), with a pixel count of 960 pixels width, 540 pixels height, spectral resolution of red, green, and blue (RGB) color, radiometric resolution of 256 levels of color depth, temporal resolution of 30 frames per second (fps; indicating the frame rate), and exposure time of 30–120 s. A microscopic movie was captured as frames and provided as images, resulting in the handling of sequential images. Based on the raw image, the analyzed target region was provided as 380 pixels width and 370 pixels height, which is the maximum width and height to completely include all of the oocytes, respectively (Figure 2).

### 2.3. Feature Vectors

First, the images generated the feature vectors using LBP, a nonparametric approach to classification. Each pixel in the image binarized with a gray-scale value of 0 to 255 was compared with the neighborhood pixels in clockwise/counterclockwise in order. For a pixel value greater than the center, the value of 0 was encoded; otherwise, the value was 1. The LBP feature vector is represented by the following:

(1) $$LBP\left(x_{c},y_{c}\right)=\overset{N-1}{\underset{i=0}{\sum }}g\left(p_{i}-p_{c}\right)2^{i}\;if\;g\left(p_{i}-p_{c}\right)\geq 0;\;g\left(p_{i}-p_{c}\right)=1,\;else;\;g\left(p_{i}-p_{c}\right)=0$$

where *N* is the sum of pixels surrounding the central pixels on a circular neighborhood, $p_{i}$ is the intensity value of the neighborhood pixel with index, and $p_{c}$ is the intensity value of the central pixel.

This study provides the number of pixel neighborhood, *N* = 8. Therefore, the histogram for all pixels of the eight-digit binary number leads to a 256-dimensional feature vector.

Second, we performed PCA for dimensionality reduction, which contributes to data compression. PCA is a multivariate analysis method that reduces information to a lower-dimensional space while preventing damage, as much as possible, to the information of multidimensional data [20,21]. This method enables many quantitative explanatory variables to be summarized into fewer indicators and synthetic variables. The feature vector $x_{n}$ is represented by:

(2) $$x_{n}={\left(x_{n1},x_{n2},x_{n3},\cdots x_{nK}\right)}^{T},\;n=1,2,\ldots ,N$$

where N is the sum number of input images and K is the number of feature values. The mean of vector m and the mean of covariance matrix S are represented by:

(3) $$m=\frac{1}{N}\overset{N}{\underset{n=1}{\sum }}x_{n}$$

(4) $$S=\frac{1}{N}\overset{N}{\underset{n=1}{\sum }}(x_{n}-m){\left(x_{n}-m\right)}^{T}$$

In the distribution of the input data in the feature vectors, the principal component is first defined by a straight line that passes through the mean and is in the direction of the largest spread. Next, the second principal component is defined by a straight line orthogonal to the first principal component, passing through the mean and in the direction of the second largest spread. Passing through the mean and in the direction of the large spread allows for the detection of the eigenvectors of the covariance matrix S. Therefore, using the covariance matrix S, the eigenvectors $u_{j}$ and the eigenvalues $\lambda_{j}$ are calculated by:

(5) $$Su_{j}=\lambda_{j}u_{j}$$

The selected number of eigenvectors $u_{j}$ in descending order is “d”, and the principal components with the number of dimensions “d” is found. For example, the eigenvectors $u_{1}$ and $u_{2}$ indicate the first and second components, respectively. In our experiments, we calculated the feature extractions using Python (Python Software Foundation, www.python.org). Python provides many libraries and algorithms for signal processing and machine learning. We used a machine with Intel Core i7-1065G7 CPU, 64 bit, 32-GB RAM.

### 2.4. SVM

SVM is one of the machine-learning models applied to classification and regression and can be used to determine the hyperplane, separating two classes of data from each other [22,23,24,25,26]. Characteristically, it includes a high generalization ability obtained by a separation hyperplane based on margin maximization, no local solution compared with conventional nonlinear models, and fast calculation by involving only a part of data. Margin maximization refers to the determination of the hyperplane identification boundary so that the distance to the support vector is maximized, which is the sample closest to the hyperplane. Nonlinear transformation is performed on the input data to obtain a separation plane in a high-dimensional space; however, the calculation quantity becomes enormous. Therefore, the calculation in the high-dimensional space is replaced by a calculation using the kernel function by the kernel trick, which avoids the mapping calculation while mapping at a higher order. The nonlinear kernel functions commonly include three kernels of Gaussian radical basis function (RBF) kernels, polynomial kernels, and sigmoid kernels. The discriminant function $f\left(x\right)$ is represented by:

(6) $$f\left(x\right)=\mathrm{sgn}\left(\overset{M}{\underset{i=1}{\sum }}\left(w_{i}\langle \Phi \left(x\right)\cdot \Phi \left(x_{i}\right)\rangle \right)+b\right)=\mathrm{sgn}\left(\overset{M}{\underset{i=1}{\sum }}\left(w_{i}K\left(x,x_{i}\right)\right)+b\right)$$

where $\mathrm{sgn}\left(z\right)$ is the signum function, $\Phi \left(x\right)$ is the nonlinear mapping function, $w_{i}$ is the vector of weight parameters, $K\left(x,x_{i}\right)$ is the kernel function, and b is the threshold. The linear kernel $K_{liner}\left(x_{1},x_{2}\right)$ and the nonlinear kernels, RBF $K_{RBF}\left(x_{1},x_{2}\right)$, polynomial function $K_{poly}\left(x_{1},x_{2}\right)$, and sigmoid function $K_{sigmoid}\left(x_{1},x_{2}\right)$, are represented by:

(7) $$K_{liner}\left(x_{1},x_{2}\right)=x_{1}^{T}x_{2}$$

(8) $$K_{RBF}\left(x_{1},x_{2}\right)=\exp\left(-\gamma \|x_{1}-x_{2}{\|}^{2}\right)$$

(9) $$K_{poly}\left(x_{1},x_{2}\right)={\left(x_{1}^{T}x_{2}+c\right)}^{d}$$

(10) $$K_{sigmoid}\left(x_{1},x_{2}\right)=\tanh\left(bx_{1}^{T}x_{2}+c\right)$$

where γ and d are the hyperparameters and c is the threshold. Finally, we evaluate our prediction model using a cross-validation method [27,28].

In the classification results, we represented the confusion matrix as true positive (TP; correctly predicted the ruptured oocyte as the ruptured oocyte), true negative (TN; correctly predicted the nonruptured oocyte as the nonruptured oocyte), false positive (FP; incorrectly predicted the nonruptured oocyte as the ruptured oocyte), and false negative (FN; incorrectly predicted the ruptured oocyte as the nonruptured oocyte). Accuracy was defined as the ratio of correctly predicted outcomes among all the frames sampled from the ruptured and nonruptured oocytes. Sensitivity was defined as the ratio of correctly predicted outcomes among all the frames sampled from the ruptured oocytes. Specificity was defined as the ratio of correctly predicted outcomes among all the frames sampled from the nonruptured oocytes. The accuracy, sensitivity, and specificity were considered as TP + TN derived from total number in this study (TP + TN + FP + FN), TP derived from TP + FN, and TN derived from TN + FP, respectively. The comparisons of ruptured and nonruptured oocytes is performed using the chi-squared test for categorical valuables [29,30,31]. Statistically significant was considered as *p* value < 0.05. Statistical data analyses were conducted with JMP Pro 16.0 (SAS Institute Inc., Cary, NC, USA).

## 3. Results

We evaluated the effectiveness of using the image texture to assess the oocyte cytoplasmic membrane before puncturing. This experiment provided microscopic movies of the ruptured oocytes (*n* = 38) and nonruptured oocytes (*n* = 55) based on the presence of membrane rupture during membrane extension. 

The legends of N (from 1 to 5) are the numbers of sampling frames. SVM is applied to classify some dimensionality data from 1 to 11: (A) Linear kernel. (B) Nonlinear kernel of RBF. (C) Nonlinear kernel of polynomial function. (D) Nonlinear kernel of sigmoid function.

On the images that were captured frame by frame in the microscopic movie, we used the sampling frames (one to five frames by one step) from the previous frames to the first confirmed frame in chronological order, with an interval of more than five frames. This allowed the selection of a subset from within a data set to estimate the characteristics of the whole data set.

Upon extracting the feature vector, we applied LBP to a 3 × 3 pixel neighborhood. PCA provided the parameter search to classify the achievement of high accuracy on the dimensionality orders (1 to 11 dimensionality by one step). Therefore, we used 5115 data set (=93 oocytes × 5 frames × 11 dimensionality), which is the maximum data set. The evaluation provided a leave-one-oocyte-out cross-validation, in which one oocyte microscopic movie datum is extracted as a validation data set, and the remaining microscopic movie data are used as a training data set. This enables to avoid including one oocyte datum in both the testing data set and the training data set, although one oocyte used some frames in this experiment. One oocyte using one frame is the same as leave-one-out cross-validation.

**Figure 3:**
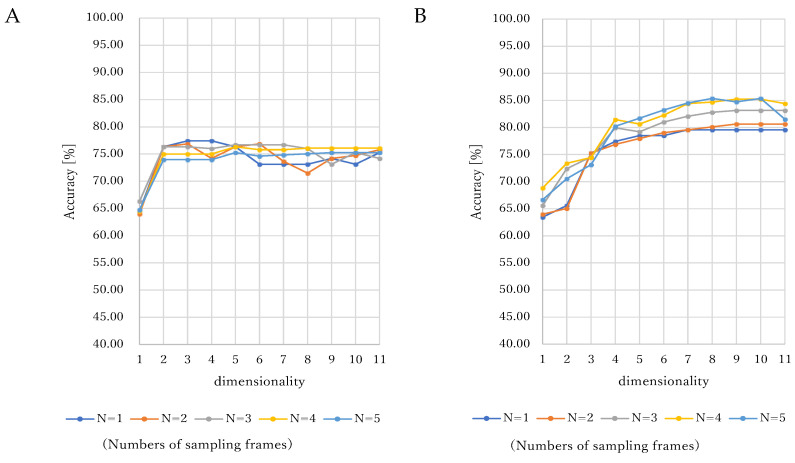

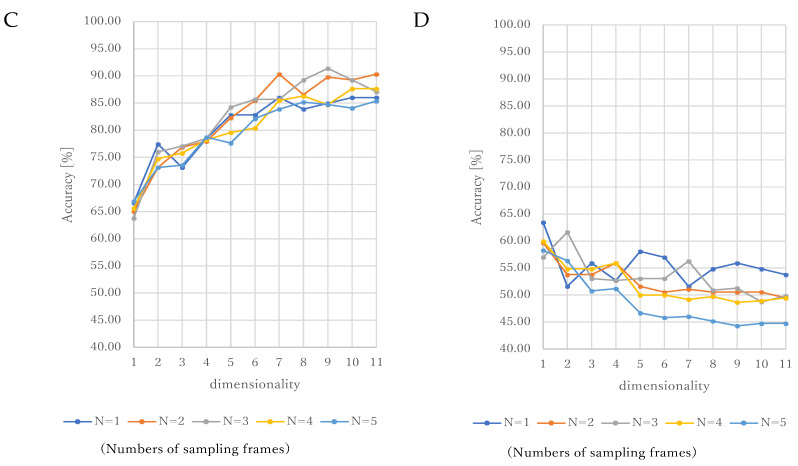
Evaluation results of SVM using four kernels. (**A**) Linear function. (**B**) Nonlinear RBF. (**C**) Non-linear poly function. (**D**) Nonlinear sigmoid function.

Figure 3 shows our experimental results for leave-one-oocyte-out cross-validation in SVM using four kernels of linear (Figure 3A), nonlinear RBF (Figure 3B), nonlinear poly function (Figure 3C), and nonlinear sigmoid function (Figure 3D). We evaluated the accuracy, sensitivity, and specificity of the classification of the trained hyperplane. As shown in Figure 3C, this method allowed us to achieve overall high classification accuracy by SVM using the nonlinear kernel of the poly function. In this SVM evaluation of the accuracy with a nonlinear kernel of poly function, we investigated the top three highest classification accuracies: 91.4% (number of sampling frames: 3, dimensionality: 9), 90.32% (number of sampling frames: 2, dimensionality: 7), and 90.32% (number of sampling frames: 2, dimensionality: 11). We confirmed a statistically significant *p* value < 0.05 in the chi-square test by calculating the confusion matrix for assessment of classification with the highest accuracy.

Table 1 shows the classification accuracy, sensitivity, and specificity of two cases of two and three sampling frames in nonlinear poly function SVM. A larger dimensionality was confirmed to lead to higher performance of accuracy. Table 2 shows the mean and standard deviation (SD) of the classification accuracy by sampling frames, and the amount of change indicates the value compared with the smaller value of the sampling frames. Table 3 shows the mean and SD of the classification accuracy by dimensionality in the feature vector of SVM, and the amount of change indicates the value compared with one smaller value for the sampling frames.

## 4. Discussion

The findings of this study underscore the fact that AI might affect the outcome of infertility care. This study showed that SVM with the feature vectors based on LBP and PCA estimated the potential for oocyte rupture. These findings are useful for developing new strategies to improve the success rate of Piezo-ICSI and new biomarkers to predict oocyte quality. To our knowledge, this is the first report to find that AI can predict the possibility of rupture of the oocyte cytoplasmic membrane based on the characteristics of a microscopic movie of the oocyte before puncturing. Of particular note is that the texture of the oocyte image before puncturing was associated with the possibility of rupture or nonrupture. The results consist of the evaluation using all the ruptured and nonruptured oocytes data as shown in Table 1, Table 2 and Table 3. The high classification performance could be achieved by considering the sampling frames, and the dimensionality and kernels in SVM. It indicates that they contain the valuable characteristic data regarding the difference between the ruptured and nonruptured oocytes. Although how much each feature affects classification performance had not been adequately investigated yet, the more detailed investigations might support the analysis techniques of the rupture discrimination. Investigating the impact of each feature on the classification and developing analytical systems for more sophisticated and personalized medicine would be part of the future work.

The possibility for rupture after ICSI might relate to the level of skills of the operators. It is reported that it may be only partially true, though the ICSI protocol seems well established [9]. Extensive training is needed to achieve more success rates, however, there remains some ethical problematic issues to provide the human oocytes as the training materials. It is expected that a medical simulator for ICSI could be developed in the future. On the other hand, we have the first report that the feature characteristics of the membrane could be a predictor to avoid the rupture [16,17,18,19]. This technique visualized the optimal puncture point with image processing of LBP in the clinical study. It is suggested to be more useful to integrate the techniques to target the membrane and target the oocyte itself.

The next step, we think, is to realize this technology in real time while performing piezo-ICSI. If the prediction is negative, our system can propose the embryologists to proceed more cautiously before the injection in that particular oocyte. In it, the combination of the technique visualized with the optimal puncture point with image processing of LBP would be required. Furthermore, by calculating the probability of the oocytes rupture, it has the potential to become an important feedback factor for improving the process of ICSI. We hope to provide a more efficient and useful puncture process.

### 4.1. Consideration of Sampling Frames

The recording time depends on the length of the microscopic oocyte movies, which in this experiment, was 30–120 s. The movie was provided in captured frames, and some frames were selected before puncturing. The sampling frames (i.e., the selected frame number) must be unified to prevent selection bias. We hypothesized that the frame data of the microscopic oocyte movie before puncturing might include important information for classifying ruptured and nonruptured oocytes. This experiment investigated the sampling frames of 1 to 5 (*N* = 1, 2, 3, 4, 5) on one microscopic oocyte movie. In Table 2, on each kernel of one linear kernel and three nonlinear kernels of RBF, polynomial function, and sigmoid function, the mean and SD of the classification accuracy for the dimensionalities of 1–11 were 74.88% ± 3.06% on three sampling frames, 80.45% ± 5.66% on four sampling frames, 82.54% ± 8.08% on three sampling frames, and 53.41% ± 3.68% on one sampling frame, respectively. The best scores had fewer than four sampling frames on all kernels. In this experiment, we found little increase in the classification performance of five sampling frames compared with four sampling frames. In our recording protocol, the temporal resolution was 30 fps and the interval was approximately five frames. Thus, the data of sampling frames within approximately 1 s before puncturing involve all of the important information for classification. Although it might be suggested that more frame images should be used for classification because including only a few frames and the numbers of sampling frames did not significantly affect performance, more sampling frames demand a larger processing cost, which is not suitable for real medical diagnosis support systems. Therefore, we concluded that five sampling frames, which equate to approximately 1-s of data, are adequate for empirically classifying ruptures and nonruptured oocytes.

### 4.2. Consideration of Dimensionality and Kernels in SVM

In this study, we hypothesized that dimensionality data not exceeding 10 might be considered to be an important determinant and predictor of classifying ruptured and nonruptured oocytes. In this experiment, we investigated the dimensionalities of 1 to 10. In Table 3, on each dimensionality of 1 to 10, the mean and SD of classification accuracies for one linear kernel and three nonlinear kernels of RBF, polynomial function, and sigmoid function were 76.20% ± 0.54% on dimensionality, 82.79% ± 2.63% on 10 dimensionality, 87.25% ± 2.22% on 10 dimensionality, and 59.67% ± 2.42% on 1 dimensionality, respectively. We added the result of 11 dimensionalities to this table because of the top score in 10 dimensionality results for nonlinear kernels of RBF and poly function. On the nonlinear kernel of RBF, the amount of change between 10 to 11 dimensionality was −0.94%, compared with 0.13% for the amount of change between 9 to 10 dimensionality. On the nonlinear kernel of the polynominal function, the amount of change between 10 to 11 dimensionality was 0.04%, as compared with 0.14% for the amount of change between 9 to 10 dimensionality. Our results with both decreases suggest that 10 dimensionality data might be the limit for predicting the possibility of oocyte rupture. Again, although it might be suggested that more dimensionalities should be used for classification, this would demand a larger processing cost, which does not result in the realization of a medical diagnosis support system. Thus, dimensionality data not exceeding 10 would better serve feature vectors for classifying ruptured and nonruptured oocytes.

### 4.3. Sample Imbalance and Size

The possibility is that a sample size imbalance on the numbers of the ruptured and nonruptured oocytes might influence the analysis for the reasons of no sufficient collection data. In this experiment, the numbers of the ruptured oocytes are smaller than the numbers of the nonruptured oocytes, which is equivalent almost to two-thirds. In generally, SVM seems to perform best with the balanced numbers, the ruptured and nonruputured oocytes in this study. However, our study utilized all the samples except the testing data set for our first step to investigate the effectivity of the proposed methods. In the future, we need to register suitable samples for the ruptured and nonruptured oocytes. In the future, we will collect more data and control the balance of data samples using the random sampling or the propensity score matching for selecting suitable data samples for training to accurately classify them into the groups of the ruptured and nonruptured oocytes.

### 4.4. Study Limitation

Our study had a relatively small sample size, especially of the ruptured oocytes. We provided the experimental design with the statistical inference to determine whether the founding results are true or incidentally. In this study, all the samples excluding the testing data set were used to investigate the effectiveness of the proposed method as the first stage. The data statistical interpretation was realized by confirming with a statistically significant *p*-value < 0.05 in the chi-square test to calculate the confusion matrix for assessment of classification. The findings of this study should be interpreted with caution due to the uncertainties regarding baseline data. Future studies should investigate larger sample sizes of oocytes. Although there is a need to explain the physiological mechanism of oocytes, it is important that findings lead to many clinical benefits. Further research is essential to determine the relationship between the possibility of rupture and microscopic frame images and to understand the mechanisms behind the oocytes.

## 5. Conclusions

In summary, in this study, we found a difference in the image texture of the microscopic movie frames between the ruptured oocytes and nonruptured oocytes. This is the first report, to our knowledge, to find that image processing enables the prediction of whether the oocyte cytoplasmic membrane has a tendency to rupture on Piezo-ICSI. Of particular emphasis is the fact that movie frames can be used as a basis for predicting the rupture possibility for the oocyte cytoplasmic membrane.

## Figures and Tables

**Table 1:** Classification accuracy in nonlinear kernel of polynomial function in SVM ^a^.

Sampling Frames	Dimensionality	Accuracy (%)	Sensitivity (%)	Specificity (%)
2	1	65.05	62.86	100.00
	2	73.12	69.23	93.33
	3	76.88	73.76	86.67
	4	77.96	79.49	75.36
	5	82.26	84.07	79.45
	6	85.48	90.29	79.52
	7	90.32	89.66	91.43
	8	86.56	88.29	84.00
	9	89.78	90.99	88.00
	10	89.25	91.67	85.90
	11	90.32	91.82	88.16
3	1	63.80	62.03	100.00
	2	75.99	71.12	100.00
	3	77.06	73.06	91.67
	4	78.49	76.65	82.93
	5	84.23	83.06	86.46
	6	85.66	87.43	83.04
	7	85.66	88.82	81.36
	8	89.25	91.41	86.21
	9	91.40	91.23	91.67
	10	89.25	89.02	89.62
	11	87.10	89.57	83.62

^a^ The bold means the maximum.

**Table 2:** Comparison of sampling frames (mean and SD) of classification accuracy in one linear kernel and three nonlinear kernels ^a^.

Sampling Frames	Mean and SD of Accuracy (Amount of Change; %)							
	Liner Kernel		Nonliner Kernel					
			RBF		Polynomial Function		Sigmoid Function	
1	74.00 ± 3.59	―	76.05 ± 5.87	―	80.74 ± 6.26	―	55.43 ± 3.38	―
2	74.05 ± 3.72	(0.05)	76.34 ± 6.10	(0.29)	82.45 ± 8.31	(1.71)	52.49 ± 3.06	(−2.93)
3	74.88 ± 3.06	(0.83)	78.82 ± 5.69	(2.48)	82.54 ± 8.08	(0.08)	53.41 ± 3.68	(0.91)
4	74.71 ± 3.42	(−0.17)	80.45 ± 5.66	(1.63)	80.55 ± 6.79	(−1.99)	51.96 ± 3.78	(−1.45)
5	73.84 ± 3.07	(−0.87)	79.73 ± 6.56	(−0.72)	79.57 ± 6.17	(−0.98)	48.54 ± 4.93	(−3.41)

^a^ The amount of change means the value compared with one smaller value for sampling frames. The bold means the maximum.

**Table 3:** Comparison of dimensionality (mean and SD) of classification accuracy in one linear kernel and three nonlinear kernels ^a^.

Dimensionality	Mean and SD of Accuracy (Amount of Change; %)							
	Linear Kernel		Nonlinear Kernel					
			RBF		Polynomial Function		Sigmoid Function	
1	64.81 ± 0.88	―	65.70 ± 2.16	―	65.60 ± 1.26	―	59.67 ± 2.42	―
2	75.60 ± 1.08	(10.79)	69.39 ± 3.86	(3.70)	74.87 ± 1.86	(9.28)	55.64 ± 3.77	(−4.03)
3	75.92 ± 1.41	(0.32)	74.53 ± 0.88	(5.14)	75.28 ± 1.85	(0.41)	53.66 ± 1.96	(−1.98)
4	75.32 ± 1.42	(−0.61)	79.18 ± 1.95	(4.65)	78.38 ± 0.29	(3.09)	53.68 ± 2.13	(0.01)
5	76.20 ± 0.54	(0.89)	79.61 ± 1.55	(0.43)	81.30 ± 2.65	(2.92)	51.88 ± 4.20	(−1.80)
6	75.43 ± 1.57	(−0.77)	80.80 ± 2.03	(1.20)	83.29 ± 2.26	(2.00)	51.28 ± 4.12	(−0.60)
7	74.82 ± 1.48	(−0.60)	82.03 ± 2.45	(1.23)	86.27 ± 2.41	(2.98)	50.84 ± 3.74	(−0.44)
8	74.35 ± 1.98	(−0.48)	82.51 ± 2.62	(0.48)	86.23 ± 2.00	(−0.05)	50.23 ± 3.45	(−0.60)
9	74.57 ± 1.13	(0.22)	82.66 ± 2.48	(0.16)	87.11 ± 3.23	(0.88)	50.13 ± 4.21	(−0.10)
10	74.89 ± 1.10	(0.32)	82.79 ± 2.63	(0.13)	87.25 ± 2.22	(0.14)	49.56 ± 3.65	(−0.58)
Additionally								
11	75.32 ± 0.72	(0.43)	81.86 ± 1.94	(−0.94)	87.29 ± 1.91	(0.04)	49.45 ± 3.20	(−0.11)

^a^ The amount of change means the value compared with one smaller value for sampling frames. The bold means the maximum.

## Data Availability

Not applicable.
